# Constructed Wetland Revealed Efficient Sulfamethoxazole Removal but Enhanced the Spread of Antibiotic Resistance Genes

**DOI:** 10.3390/molecules25040834

**Published:** 2020-02-14

**Authors:** Shuai Zhang, Yu-Xiang Lu, Jia-Jie Zhang, Shuai Liu, Hai-Liang Song, Xiao-Li Yang

**Affiliations:** 1 Jiangsu Key Laboratory of Atmospheric Environment Monitoring and Pollution Control (AEMPC), Collaborative Innovation Center of Atmospheric Environment and Equipment Technology (CIC-AEET), Nanjing University of Information Science &Technology, Nanjing 210044, China; zhangshuai198702@163.com; 2 School of Environment, Nanjing Normal University, Jiangsu Engineering Lab of Water and Soil Eco-remediation, Wenyuan Road 1, Nanjing 210023, China; lu_uyx@163.com; 3 School of Civil Engineering and Architecture, East China Jiaotong University, Nanchang 330013, China; zhangjiajieedu@163.com; 4 College of Environment and Safety Engineering, Qingdao University of Science and Technology, Qingdao 266042, China; liushuai@qust.edu.cn; 5 School of Civil Engineering, Southeast University, Nanjing 210096, China

**Keywords:** sulfamethoxazole, antibiotic resistance genes, *sul* genes, bacterial community, constructed wetlands

## Abstract

Constructed wetlands (CWs) could achieve high removal efficiency of antibiotics, but probably stimulate the spread of antibiotic resistance genes (ARGs). In this study, four CWs were established to treat synthetic wastewater containing sulfamethoxazole (SMX). SMX elimination efficiencies, SMX degradation mechanisms, dynamic fates of ARGs, and bacterial communities were evaluated during the treatment period (360 day). Throughout the whole study, the concentration of SMX in the effluent gradually increased (*p* < 0.05), but in general, the removal efficiency of SMX remained at a very high level (>98%). In addition, the concentration of SMX in the bottom layer was higher compared with that in the surface layer. The main byproducts of SMX degradation were found to be 4-amino benzene sulfinic acid, 3-amino-5-methylisoxazole, benzenethiol, and 3-hydroxybutan-1-aminium. Temporally speaking, an obvious increase of *sul* genes was observed, along with the increase of SMX concentration in the bottom and middle layers of CWs. Spatially speaking, the concentration of *sul* genes increased from the surface layer to the bottom layer.

## 1. Introduction

In recent years, antibiotics have been extensively used as livestock food additives and to fight infections in animal husbandry [1,2]. The overuse of antibiotics results in their continuous release into the environment in China. Additionally, previous investigations have indicated that antibiotics in animal waste could not be entirely removed using traditional lagoon treatment and in wastewater treatment plants (WWTPs) [3]. As a result, antibiotics were widely detected in wastewater, surface water, and groundwater [4,5].

The wide presence of antibiotics in the environment could cause concern because it not only causes serious toxic effects on organisms, but also promotes the spread of antibiotic-resistant genes (ARGs) [6], even with low concentrations in the environment [7,8]. ARGs could be spread through horizontal gene transfer (HGT) and vertical gene transfer (VGT), and in many cases, could be maintained in microbial populations, even without selection pressure from antibiotics [9,10,11]. HGT is a major pathway for the transfer of ARGs, including conjugative transposons, integrons, insertion sequences, and plasmids [11,12]. ARGs have often been detected as part of antibiotic resistance super integrons. Therefore, one antibiotic may coselect resistance to other antibiotics when applying multiple antibiotics [13]. Even if antibiotic-resistant bacteria were damaged or killed, ARGs could still be released to the environment and then transformed into other bacteria [14,15]. Previous studies have revealed the high relative abundances of ARGs in wastewater lagoons and municipal wastewater, even after treatment [16,17,18]. In recent years, ARGs were regarded as fast-growing potential pollution because of the extensive application of antibiotics in the livestock industry [19,20,21]. Hence, effective treatment processes for antibiotic removal could also prevent the spread of ARGs.

Constructed wetlands (CWs) are designed and constructed exploiting natural processes to treat rural wastewater [22,23]. The advantages of CWs mainly lie in high purification removal, relatively low construction and maintenance costs, reduced energy consumption, convenient operation, and broad application prospects in developing countries or rural areas [24,25]. Recently, CWs were used to remove antibiotics from agricultural and municipal wastewater via natural processes involving plants, soil/sediment, and microorganisms [21,26,27]. CWs have been shown to be more efficient in the removal of antibiotics and ARGs than conventional wastewater treatment systems [16,28]. Liu et al. (2013) [28] found that the total absolute abundances of tetracycline resistance (*tet*) genes and the 16S rRNA were reduced by 50% from swine wastewater using CWs. Huang et al. (2015) [16] reported that the absolute abundances of the ARGs were greatly reduced, with their log units ranging from 0.26 to 3.3. However, previous studies of ARG reduction have always focused on their elimination, rather than the induction of ARGs along with antibiotics removal by CWs. CWs could also be a significant source of ARGs, and may enhance their spread. Therefore, exploring the induction of ARGs in CWs would greatly assist in evaluating their environmental risks.

Sulfamethoxazole (SMX) is a synthetic antibiotic within the sulfonamide antibiotic family, and is largely consumed in the livestock husbandry [29,30]. *Sul* genes (*sulI* and *sulII*) were chosen as the representatives of ARGs for their frequent use [17,31,32,33]. In this study, four CWs were established to treat a synthetic wastewater containing SMX for 360 day. The objectives of this study were: (1) to investigate the elimination efficiencies and products of SMX, (2) to explore the development of *sul* genes in the reactors, (3) to assess the risks of ARGs in effluent, and (3) the bacterial community during the treatment process.

## 2. Results and Discussion

### 2.1. SMX Removal Efficiency

**Figure 1:**
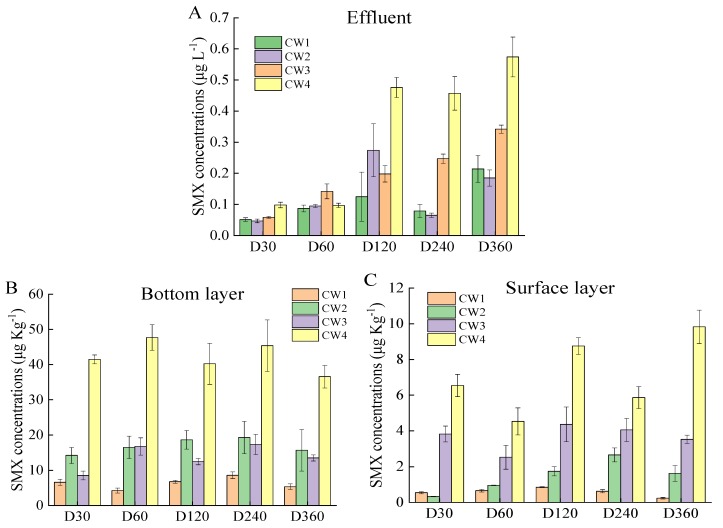
Concentrations of SMX (mean ± SD, *n* = 3) in the effluent water and layers. (**A**) concentration of SMX in the effluent; (**B**) concentration of SMX in the bottom layer; (**C**) concentration of SMX in the surface layer.

The concentrations of SMX in the effluent, bottom layer, and surface layer of CWs at five sampling points are shown in Figure 1. The concentrations of SMX in the effluent ranged 0.051–0.214, 0.047–0.274, 0.0584–0.342, and 0.098–0.574 μg L^−1^ in CW1, CW2, CW3, and CW4. CW4 fed with 200 μg L^−1^ SMX exhibited significantly higher concentrations of SMX in the effluent than CW1 fed with 20 μg L^−1^ (*p* < 0.05). On D360, the SMX concentrations in the effluent were 0.214, 0.185, 0.342, and 0.574 μg L^−1^ with influent SMX concentration of 20, 50, 100, and 200 μg L^−1^, respectively. Temporally speaking, the SMX concentrations in effluent gradually increased during the study (*p* < 0.05). In the effluent of CW3, SMX concentrations were 0.0584, 0.142, 0.198, 0.247, and 0.342 on D30, D60, D120, D240, and D360, respectively. Notably, excellent removal efficiencies (>98%) for SMX were obtained using CWs, even when the influent SMX concentration was as high as 200 μg L^−1^.

SMX has been reported to be easily biodegradable, especially under anaerobic conditions in the CWs [25]. In the present study, it was noteworthy that the removal efficiencies of antibiotics by different CWs were even better than conventional WWTPs [34]. As demonstrated by previous experiments, biodegradation, absorption, hydrolysis, and photodecomposition played significant roles in the removal of antibiotics in the solid and aqueous phases in CWs [34,35,36].

Previous reports also showed that the adsorption process only accounted for a minor percentage of the total removal in CWs [12].

The SMX concentrations of the bottom layer ranged 4.256–8.620, 14.213–19.281, 8.546–17.322, and 36.564–47.684 μg Kg^−1^ in CW1, CW2, CW3, and CW4. The SMX concentrations of the surface layer ranged 0.239–0.852, 0.327–2.652, 2.526–4.365, and 4.531–9.829 μg Kg^−1^ in CW1, CW2, CW3, and CW4, respectively. The difference of SMX concentration between the bottom and surface layers was affected by the influent SMX concentration. This was probably because the relatively high *K_d_* values prompted SMX to be adsorbed in the substrates of CWs [37]. However, the SMX concentrations in the layers did not significantly increase during the treatment (*p* > 0.05). This result was probably due to the fact that other physicochemical and biological processes occurred jointly or separately after SMX adsorption in substrate [16]. Clearly, the concentration of SMX in the bottom layer was higher compared with that in surface layer. This was not surprising, because that the bottom layer was faced with a higher concentration of SMX due to the vertical up-influent.

### 2.2. SMX Degradation Products

**Figure 2:**
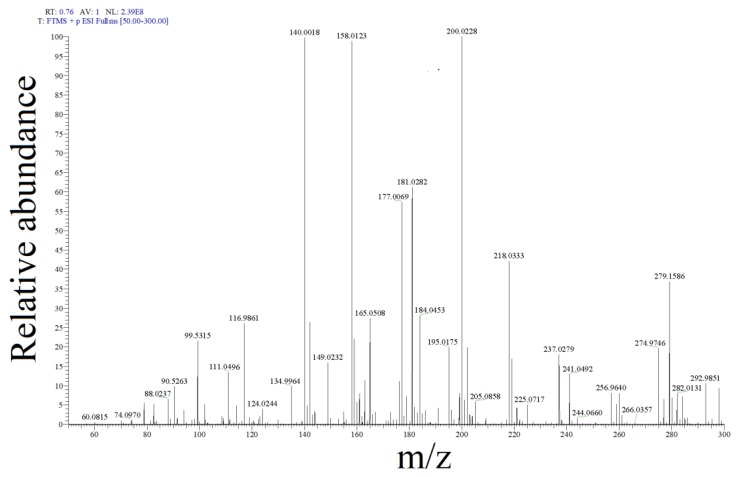
Q-Exactive spectrum degradation products of SMX.

LC-MS/MS was employed to identify the degradation products in order to clarify the degradation pathway of SMX (C_10_H_11_N_3_O_3_S, *m*/*z* 252.0450). According to the detected mass/charge ratios under positive mode, several potential metabolites were identified, including 4-amino benzene sulfinic acid (C_6_H_7_NO_2_S, *m*/*z* 158.0123), 3-amino-5-methylisoxazole (3A5MI, C_4_H_6_N_2_O, *m*/*z* 99.5315), benzenethiol (C_6_H_6_S, *m*/*z* 111.0496), and 3-hydroxybutan-1-aminium (C_4_H_11_NO, *m*/*z* 90.5263) (Figure 2). A previous report indicated that the removal of antibiotics was mainly caused by adsorption rather than degradation in wastewater treatment plants [38]. However, in this study, it was found that the products of SMX may be transformed by bacterial communities. A previous study also found 4-amino benzene sulfinic acid and 3A5MI during the degradation of SMX [39]. During the degradation process, SMX was initially hydrolyzed into 4-amino benzene sulfinic acid and 3A5MI; then, 4-amino benzene sulfinic acid was further transformed into benzenethiol, which was finally degraded to generate methane or carbon dioxide. During the transformation of 3A5MI to 3-hydroxybutan-1-aminium, the isoxazole ring was opened and its nitrogen atom was removed. Since 3-hydroxybutan-1-aminium has a chain structure, it can be easily mineralized into methane by microbes under anoxic conditions [40].

### 2.3. Sul Genes in the Effluent and Media

**Figure 3:**
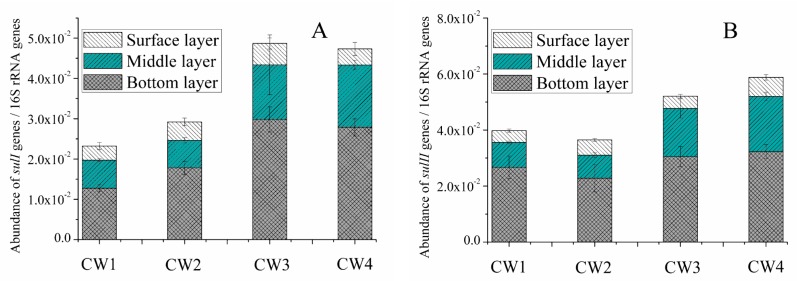
*Sul* genes (*sulI* and *sulII*) normalized to 16S rRNA genes in the layers (bottom layer, middle layer and surface layer) of CW1, CW2, CW3, and CW4, respectively (mean ± SD, *n* = 3). (**A**) *sulI* genes; (**B**) *sulII* genes.

In order to analyze the changes of corresponding target genes, the relative abundances of *sul* genes were analyzed during the treatment process. The distributions of two *sul* (*sulI* and *sulII*) in the CWs are shown in Figure 3. The relative abundances of *sul* genes showed an obvious increase with the increase of SMX concentrations in the bottom and middle layers. In wastewater treatment installations, the abundances of ARGs were not only determined by their abundances, but were also affected by the concentrations of antibiotics in the influent [6]. Since synthetic wastewater was used in this study, the abundances of *sul* genes in the influent may be ignored. In addition, the *sul* genes were not found in CW0. Therefore, most of the target genes were induced by the antibiotics in the influent. Further, bacteria would also gain the corresponding ARGs via VGT and HGT [10].

Notably, the concentration of *sul* genes in the bottom layer was higher than that in the middle layer, with the surface layer containing the least (Figure 3). A similar result was obtained in a previous study, which found that the level of ARGs was high in CWs [28]. This observation was mainly influenced by the level of SMX sources and oxygen transport capacity in the bottom layer [28]. Clearly the concentration of SMX in the bottom layer was the highest, followed by the middle and surface layers (Figure 1). The fate of ARGs in the different layers of CWs was mainly attributable to the accumulation of SMX in the substrate. Interestingly, the relative abundances of *sul* genes in the CWs were in the order of *sulII* > *sulI* (Figure 3). This was not surprising, because the different fates of *sul* genes in CWs were mainly caused by their specific mechanisms [6]. The *sulI* gene was generally found to be associated with other ARGs in class 1 integrons, while *sulII* was usually located on small nonconjugative plasmids, or generally located on large transmissible multi-resistance [41]. Therefore, the persistence of *sulII* genes might be attributed to the successive pressure exerted by antimicrobial agents that were transferred via HGT and VGT between pathogens, nonpathogens, and even distantly related organisms [31,41].

**Figure 4:**
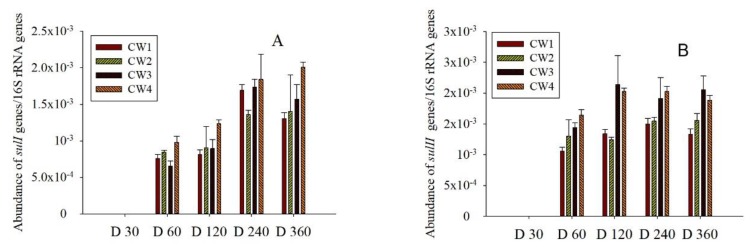
*Sul* genes (*sulI* and *sulII*) normalized to 16S rRNA genes in the effluent of CW1, CW2, CW3, and CW4, respectively (mean ± SD, *n* = 3). (**A**) *sulI* genes; (**B**) *sulII* genes.

ARGs, as a major source of pollution, may be spread in bioreactors [42]. The relative abundances of corresponding *sul* genes in effluent have been reported (Figure 4). The CWs, indicating the rate of spread for *sul* genes, were probably caused by antibiotics. This was comparable to previous reports in which similar abundances of ARGs were observed in the effluent of CWs [21]. In addition, the relative abundance of most *sul* genes was enhanced with higher concentrations of SMX; *sul* genes were not detected in the effluent of CW0. The relative abundance of *sul* genes exhibited an increase, which tended to be stable among the treatment duration. Meanwhile, the relative abundances of *sul* genes in the effluent were in the order of *sulII* > *sulI*. In a word, vertical up-CWs may be a good choice for application as an effective SMX removal method, but the fate of ARGs remains to be further studied in practical applications.

### 2.4. Composition of Bacterial Communities

**Figure 5:**
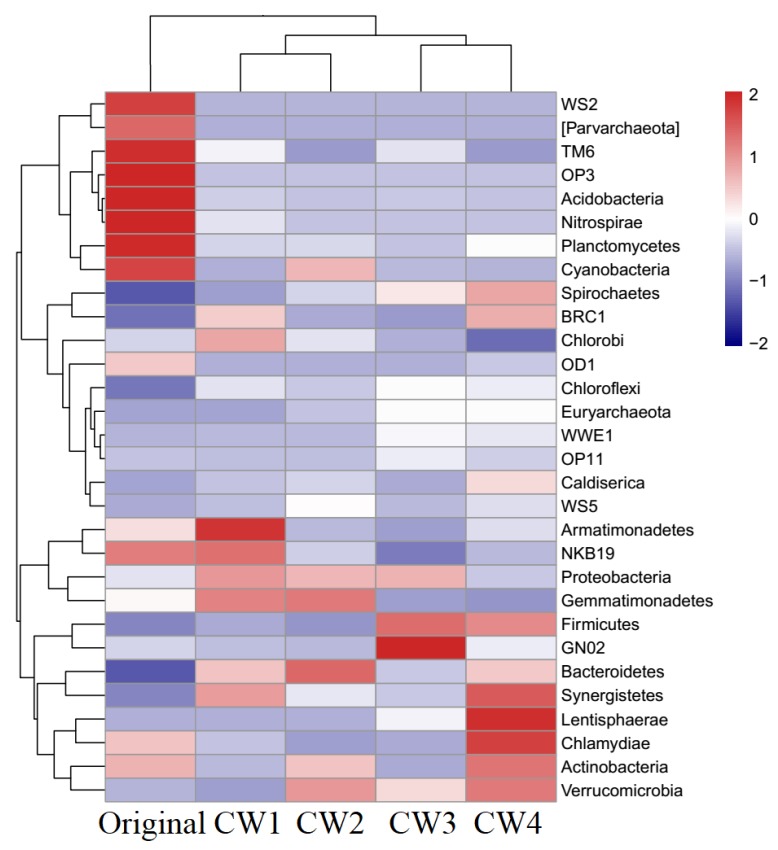
Heat map of bacterial populations in phylum level: Sample names were listed on the *x*-axis, and genus names were listed on the *y*-axis.

The microbial community was evaluated in terms of abundance and bacterial structure in response to the different treatments in CWs [43]. There were 30 dominant genera in the phylum level for the bottom layers, occupying > 94.90% of sequences (Figure 5). Twenty genera were identified while 10 remained unknown by taxonomy assignment. *OD1* (49.32%), *Proteobacteria* (44.59%), *Proteobacteria* (31.36%), and *Proteobacteria* (54.53%) had the maximum amounts of dominant phylum and high relative abundances of detection in CW1, CW2, CW3, and CW4, respectively, followed by *Chloroflexi*, *Bacteroidetes,* and *Acidobacteria*. Previous studies reported that proteobacteria is responsible for the degradation of refractory organic [44]. In addition, *Proteobacteria* (12.79%), *Bacteroidetes*, *Acidobacteria,* and *Planctomycetes* increased after the SMX treatment process. Because of potential coselection on SMX, the accumulation of antibiotics in substrates may also contribute to the enhancement of some dominant bacteria. Our result was similar to that in a previous report, i.e., that *Proteobacteria* occupied the string majority in antibiotic-correlated bacteria, followed by *Bacteroidetes* and *Actinobacteria* [45]. Some specific bacteria in CWs, like *Nitrospirae*, are well-known to be involved in nitrification and ammonia oxidization [46]. In accordance with our result, *Nitrospirae* was a dominant phylum, with a relative abundance of up to 4.45%.

To assess the variation of dominant bacteria after CW treatment, five samples could be divided into three clusters according to the bacterial composition and relative abundance. Cluster I comprised the original sample, cluster II included the CW1 and CW2 samples, and cluster III comprised all of the remaining samples (CW3 and CW4). Previous study has shown that no significant difference was observed in terms of bacterial abundance, richness, or diversity among different treatments of antibiotics [43]. However, in this experiment, the bacterial communities and their relative abundance were influenced by the SMX content of the influent. Therefore, the phenomenon suggested that the original bacterial structure had to adapt to the variation of different SMX concentration conditions, appearing to change over the 360 days of the experiment period.

## 3. Materials and Methods

### 3.1. Reactor Configuration

Four CWs were set up with 65 cm in height and 35 cm in diameter with the temperature maintained at 26 ± 3 °C and a relative humidity of 55–65%. The CWs were filled with siliceous gravel and sand (siliceous gravel: sand volume ratio = 1:1; siliceous gravel was 4–7 mm and sand was 5–8 mm in diameter). *Phragmites australis* were transplanted into the top layer. The synthetic wastewater was fed into four CWs from the bottom inlet by peristaltic pump with a hydraulic loading rate of 5 cm d^−1^. The synthetic wastewater was prepared with tap water containing chemical oxygen demand (500 mg L^−1^), ammonia nitrogen (40 mg L^−1^), total nitrogen (150 mg L^−1^), and total phosphorus (20 mg L^−1^). Then, 0, 20, 50, 100, and 200 μg L^−1^ of SMX were spiked into synthetic wastewater for CW0, CW1, CW2, CW3, and CW4, respectively.

### 3.2. SMX Detection

First, 1000 mL of effluent was collected at days 30, 60, 120, 240, and 360, to measure the SMX concentrations. Then, 200 g wetland media was collected from the bottom, middle, and surface layers on day 360. Both water samples and wetland media were taken in triplicate. Water samples were filtered through 0.45 µm fiber filters [16]. Wetland media were extracted by solid-phase extraction (Waters, Millford, MA, USA) [47]. Liquid chromatography-mass spectrometry (LC-MS/MS, Thermo Scientific Q Exactive Hybrid Quadrupole-Orbitrap; Thermo Fisher Scientific, Waltham, MA, USA) was used to analyze the concentrations of SMX in water and wetland media. The mobile phase was composed of pure 30% acetonitrile and 70% water solution (*v*/*v*) [39]. Hypersil GOLD C18 column (Acquity UPLC BEH C18; 100 mm × 2.1 mm, 3 μm) was used in this study. The capillary voltage was 3.8 (±) kV, the collision energy was 8 eV, and the capillary temperature was set to 350 °C. SMX products were detected by the LC-MS/MS in positive mode [16].

### 3.3. DNA Extraction and ARG Analysis

Genomic DNA was extracted from effluents (200 mL) and wetland media (5 g) using a DNA extraction kit (MoBio, Carlsbad, CA, USA) at each sampling point in Section 2.2. The DNA concentrations were tested using ND-1000 NanoDrop spectrophotometer (Thermo Fisher Scientific, Wilmington, DE, USA). The 16S rRNA gene and two *sul* genes (*sulI* and *sulII*) were quantified by a Real-Time PCR System (CFX96, Bio-Rad). The reaction was 25 μL on 96-well plates (Bio-Rad, Shanghai) containing 12.5 μL SYBR Green qPCR mix (Bio-Rad, Shanghai), 0.5 μL of each forward and reverse primers (Bio-Rad, Shanghai), 1 μL DNA templates, and 10.5 μL ddH_2_O [48]. PCR protocol and primer sequences of *sul* genes were based on previous studies [49,50].

### 3.4. High-Throughput Sequencing

Wetland media (5 g) were collected at the bottom layer to analyze microbial community. The V4 region of the bacteria 16S rRNA gene was amplified by PCR (5′-GTGCCAGCMGCCGCGGTAA-3′, 5′-GGACTACHVGGGTWTCTAAT-3′) according to previous reports [51,52]. Each PCR reaction was performed with 50 μL mixture containing 35 ng template DNA, 4 μL PCR Primer Cocktail (5 μM, Qiagen, Valencia, CA, USA), 25 μL PCR Master Mix (Qiagen), and ddH_2_O [53]. Illumina MiSeq platform was employed to purify and pool amplicons in equimolar amounts and paired-end sequenced (2 × 250) [51,54]. Data were analyzed with Microsoft Excel 2010, and statistical analyses were conducted by SPSS ver. 19.0.

## 4. Conclusions

This study clearly demonstrated that excellent SMX removal efficiency among different SMX concentrations was obtained during the treatment in the CWs. The concentration of SMX in the bottom layer was higher compared with that in the surface layer. Good removal efficiencies for SMX were observed using the systems. A degradation mechanism of SMX was proposed. The relative abundances of *sul* genes showed an obvious increase with the increase of SMX content in the bottom and middle layers. The concentration of *sul* genes in the bottom layer was shown to be higher than that in the middle layer; the surface layer presented the lowest concentration. The relative abundance of *sul* genes exhibited an increase, which tended to be stable among the treatment duration. *Proteobacteria* was the dominant phylum in the CWs.
